# lncRNA HOTAIRM1 Activated by HOXA4 Drives HUVEC Proliferation Through Direct Interaction with Protein Partner HSPA5

**DOI:** 10.1007/s10753-023-01919-x

**Published:** 2023-10-29

**Authors:** Yu Zhou, Qiang Wu, Xiangshu Long, Youfu He, Jing Huang

**Affiliations:** 1 Medical College, Guizhou University, Guiyang, 550025 Guizhou China; 2 Department of Cardiology, Guizhou Provincial People’s Hospital, Guiyang, 550002 Guizhou China

**Keywords:** long non-coding RNAs, HOTAIRM1, HOXA4, HSPA5, vascular endothelial cells, proliferation

## Abstract

Despite the substantial progress in deciphering the pathogenesis of atherosclerosis (AS), cardiovascular mortality is still increasing. Therefore, atherosclerotic cardiovascular disease remains a sweeping epidemic that jeopardizes human health. Disentangling the molecular underpinnings of AS is imperative in the molecular cardiology field. Overwhelming evidence has indicated that the recognition of a fascinating class of players, known as long non-coding RNAs (lncRNAs), provides causality for coordinating AS. However, the function and mechanism of HOTAIRM1 are still poorly understood in human umbilical vein endothelial cells (HUVECs) and AS. Herein, we primarily underscored that lncRNA HOTAIRM1 is potentially responsible for AS; as such, it was dramatically up-regulated in HUVECs upon ox-LDL stimulation. Functionally, HOTAIRM1 knockdown attenuated HUVEC proliferation and potentiated apoptosis in the absence and presence of ox‐LDL. Furthermore, HOTAIRM1 was preferentially located in the nuclei of HUVECs. Mechanistically, HOXA4 is directly bound to the HOTAIRM1 promoter and activated its transcription. Of note, a positive feedback signaling between HOXA4 and HOTAIRM1 was determined. Intriguingly, the interplay between HOTAIRM1 and HSPA5 occurred in an RNA-binding protein pattern and a transcription-dependent regulatory manner. In addition, HSPA5 overexpression partially antagonized HUVEC proliferation inhibition of HOTAIRM1 depletion. Taken together, our findings delineate a pivotal functional interaction among HOXA4, HOTAIRM1, and HSPA5 as a novel regulatory circuit for modulating HUVEC proliferation. An in-depth investigation of the HOXA4-HOTAIRM1-HSPA5 axis promises to yield significant breakthroughs in identifying the molecular mechanisms governing AS and developing therapeutic avenues for AS.

**Graphical Abstract:**

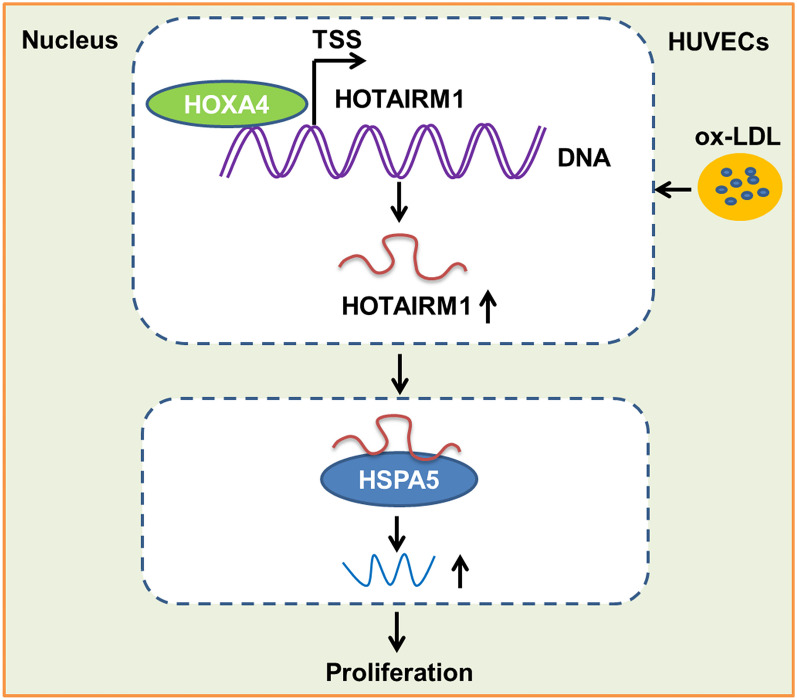

**Supplementary Information:**

The online version contains supplementary material available at 10.1007/s10753-023-01919-x.

## INTRODUCTION

Atherosclerosis (AS) is a complex and chronic inflammatory disease characterized by lipid deposition in the arterial wall that is responsible for diverse cardiovascular events [1, 2]. The aberrant vascular endothelial cell (VEC) proliferation is a hallmark of early AS and vascular injury progression [3]. Endothelial cell injury or dysfunction is the initial step in AS [4]. VECs are one of the primary cellular constituents of the blood vessel wall, and the destruction of their integrity is a pivotal factor in AS pathogenesis [5]. The dynamic equilibrium between VEC proliferation and apoptosis is the key cytological basis for maintaining VEC integrity and ensuring normal vascular function [6]. Thus, interrogating novel molecules affecting the proliferation and apoptosis of VEC is important in AS. In this study, a cellular model induced by ox-LDL was used to elucidate the mechanism underlying AS.

Long non-coding RNAs (lncRNAs) represent a broad class of non-coding RNAs that are over 200 nucleotides in length [7, 8]. Burgeoning findings have shown that lncRNAs are versatile regulators of multilevel gene expression, including transcription, post-transcriptional processes, alternative splicing, protein translation and modification, protein transport, and localization [9–13]. lncRNAs are pivotal in shaping cellular activity as functional units in various cancers, such as cell proliferation, migration, and apoptosis [14]. There is a burgeoning interest in lncRNAs because of their roles in AS development and progression [15]. For instance, the lncRNA HOXA11-AS promotes vascular endothelial cell injury in AS by regulating the miR-515-5p/ROCK1 axis [16]; lncRNA DANCR accelerates AS development by regulating the miR-214-5p/COX20 signaling pathway [17]; and lncRNA PVT1 knockdown reduces AS by inactivating the MAPK/NF-κB pathway [18]. Although no such role has yet been delineated regarding HOXA transcript antisense RNA myeloid-specific 1 (HOTAIRM1) in the cardiology field and less is known about its functional annotation and molecular underpinnings in human umbilical vein endothelial cell (HUVEC), it appears that lncRNAs can still have an impact on AS onset and progression. Therefore, deciphering the functional significance and molecular machinery of HOTAIRM1 is crucially important in this field; however, it is increasingly challenging.

HOXA transcript antisense RNA myeloid-specific 1 (HOTAIRM1), located at the 5′ end of homeobox A (HOXA) gene cluster with a length of 1052 bp, is a natural antisense transcript of HOXA1 gene exclusively expressed in the myeloid lineage [19] and is induced during neuronal differentiation [20]. HOTAIRM1 was initially discovered and investigated in NB4 promyelocytic leukemia cells, and its knockdown halted all-trans retinoid acid (ATRA)-induced granulocytic differentiation [21, 22]. More recently, it was shown to be important in multiple cancers, such as breast cancer [23], lung cancer [24], and glioblastoma multiforme [25]. Notwithstanding, the role of HOTAIRM1 in diseases has largely highlighted its implication in tumors, as to whether it is of biological significance, and whether its new perspective in HUVECs remains to be elucidated functionally and mechanistically. The primary signaling pathway regarding HOTAIRM1 refers to the NF-κB [26–28] and Wnt signaling pathways [29, 30] in various biological regulations to date. Robust data indicate that these pathways are strongly associated with AS. Therefore, we speculate that HOTAIRM1 is likely to play an important role in AS onset and progression. Further exploration is imperiously warranted, and insights into the mechanistic detail of HOTAIRM1 may shed light on a new direction in therapeutic strategies for AS.

In this study, an unprecedented role of HOTAIRM1 was corroborated in HUVECs; we determined the influence of ox-LDL on HOTAIRM1 expression in HUVECs and provided mechanistic insights into the HUVEC proliferation regulation of HOTAIRM1 by the interplay between HOXA4 and its downstream associated protein partner, HSPA5.

## MATERIALS AND METHODS

### Ethics Approval and Consent to Participate

This study was approved by the Ethics Committee of Guizhou Provincial People’s Hospital. The animal experimental protocol was approved by the Animal Ethics Committee of Guizhou Provincial People’s Hospital (License number: 2022–038).

### AS Mouse Model

Male ApoE mice in the AS group were fed a high-fat diet (21% fat and 0.15% cholesterol) at 4 weeks of age. Male ApoE mice in the control group were fed a normal diet (4% fat and 0% cholesterol). After 12 weeks, the mice were sacrificed, and the aortas were removed for further experiments.

### Cell Culture, Treatment, and Transfection

HUVECs were obtained from the American Type Culture Collection (ATCC, Manassas, VA, USA, AC337632). The cells were cultured in an F-12 K medium (Invitrogen, Carlsbad, CA, USA, 21127022) containing 10% FBS (Gibco) and 1% penicillin–streptomycin (Thermo Fisher Scientific, 15140122). All cells were grown at 37 ℃ in a humidified atmosphere containing 5% CO2. For ox-LDL induction experiments, HUVECs were induced with ox-LDL (100 μg/mL, UnionBiol, Beijing, China, jk-002) for 24 h when the cells reached 70–80% confluence. HOXA4 and HSPA5 in HUVECs were knocked down using small interfering RNAs (siRNAs), and HOTAIRM1 was silenced using antisense oligonucleotides (ASOs). The ASOs and siRNAs were synthesized by GenePharma (Shanghai, China). The pcDNA3.1 vectors from Invitrogen were used to construct pcDNA3.1/HOXA4, pcDNA3.1/HOTAIRM1, and pcDNA3.1/HSPA5, with the empty vector as the negative control. Lipofectamine 3000 (Invitrogen, L3000015) was used for the transfection experiments.

### Quantitative Real-time Polymerase Chain Reaction

Total RNA was extracted from the arterial tissue and HUVECs using TRIzol reagent according to the manufacturer’s protocol (Thermo Fisher Scientific, 12183555), and cDNA was acquired following reverse transcription. Quantitative real-time polymerase chain reaction (RT-qPCR) was performed using the SYBR Green Master Mix (Takara, Dalian, China, RR036A). The results were normalized to the expression of β-actin, and data were analyzed based on the 2 − ΔΔCt method. Supplementary File 1 lists the forward and reverse primers.

### Western Blotting

Total cellular protein was extracted using RIPA lysis buffer (Beyotime, Shanghai, China, P0013B) and subjected to sodium dodecyl sulfate–polyacrylamide gel electrophoresis. Separated proteins were transferred to PVDF membranes, treated with 5% skim milk at room temperature for 2 h, and then incubated with corresponding primary antibodies against HOXA4 (Abcam, 131049), HSPA5 (Abcam, ab108615), PCNA (CST, 13110), cyclin D1 (Abcam, ab16663), CKD4 (Abcam, ab108357), caspase-3 (Abcam, ab32351), caspase-9 (Abcam, ab32539), Bax (Abcam, ab32503), Bcl-2 (Abcam, ab185002), and GAPDH (Abcam, ab181602) overnight at 4 °C, followed by conjugation with horseradish peroxidase at 37 °C for 2 h. Band density was visualized using an ECL kit (Invitrogen, Carlsbad, CA, USA, WP20005).

### Oil Red O Staining and H&E Staining

Tissues were stained with filtered Oil Red O solution (Sigma). Oil red staining was visualized under a microscope. The root and aortic arch bifurcation vessels were immediately fixed in 4% neutral formaldehyde solution, dehydrated by soaking in 75% distilled water, and immersed in formaldehyde solution for H&E staining.

### Cell Proliferation Assays and Cell Cycle Analysis

For the Cell Counting Kit-8 (CCK-8) assay, to detect the viability of HUVECs, transfected-HUVECs were added to CCK-8 solution (Dojindo, KMJ, Japan, CK04) at the indicated time and incubated for 2 h at 37 °C; the absorbance was determined at the wavelength of 450 nm. For the EdU assay, HUVECs were incubated in a 24-well plate for transfection for 72 h and subsequently added to 100 µL of EdU (RIBOBIO, Shanghai, China, C10310-1). A fluorescence microscope was used to visualize the cells (Olympus, Tokyo, Japan). For cell cycle assay, after transfection for 72 h, cells were digested and washed with PBS and then fixed in 70% ice-cold ethanol at 4 °C overnight. The cells were treated with 500 µL of RNase A, stained with 50 μg/mL propidium iodide (PI) for 15 min, and examined by flow cytometry.

### Cell Apoptosis Assay

For apoptosis analysis, cells were double-stained with 7-AAD and PE per the manufacturer’s instructions (559763; PE Annexin V Apoptosis Detection Kit I, BD, USA, 559763). GFP-positive cells were assessed by flow cytometry (FACSCantoII, 338960; BD Biosciences, San Jose, CA, USA, 338960). Terminal deoxynucleotidyl transferase dUTP nick-end labeling (TUNEL) staining was carried out to test cell apoptosis. Briefly, the cells were fixed using 4% formaldehyde, followed by staining with the TUNEL kit according to the manufacturer’s instructions (Vazyme, TUNEL Bright-Red Apoptosis Detection Kit, A113). The TUNEL-positive cells were counted using a fluorescence microscope.

### Chromatin Immunoprecipitation Assay

Chromatin immunoprecipitation (ChIP) analysis was conducted using a Millipore ChIP Assay Kit (Millipore, MA, USA, 17–295) according to the manufacturer’s instructions. HUVECs were cross-linked with formaldehyde and sonicated at 200–1000 bp. Sonicated lysates were cleared and incubated overnight at 4 °C with magnetic beads coupled with HOXA4 antibody (Santa Cruz, sc-398426). RT-qPCR was used to analyze the precipitated chromatin DNA.

### Dual-Luciferase Reporter Assay

To dissect the transcriptional regulation of HOXA4 on HOTAIRM1, the sequence of the wild-type or indicated site-mutated HOTAIRM1 promoter (WT, Site 1-MUT, Site 2-MUT, Site 3-MUT, Site 4-MUT, and MUT) was synthesized and cloned into the pGL3 vector to construct the corresponding reporter plasmids. Thereafter, the indicated recombinant reporter was co-transfected with an empty vector or a HOXA4 plasmid into HUVECs. After 48 h, the luciferase activity in each group was measured using a Dual-Luciferase Reporter Assay System (Promega, Madison, WI, USA).

### Fluorescence In Situ Hybridization Assay

A fluorescent in situ Hybridization (FISH) Kit was used to analyze HOTAIRM1 subcellular location (GenePharma, Suzhou, China). Cy3-labeled HOTAIRM1 probe was also synthesized. Images were acquired using a confocal microscope (Olympus). GAPDH and U6 served as controls for cytoplasmic and nuclear RNA, respectively.

### RNA Pull-Down Assay and Mass Spectrometry

Biotin-labeled sense and antisense HOTAIRM1 sequences were obtained using the Transcript Aid T7 High-Yield Transcription Kit (Thermo Scientific, K0441). A MEGAclearTM Kit (Thermo Scientific) was used to recycle the sequences per the manufacturer’s instructions. The sequences were incubated with cell lysates for 4 h at 4 °C, and then the biotin-labeled RNAs and their binding protein partner were pulled down by streptavidin magnetic beads (Thermo, USA, 20164) at 4 °C overnight. Proteins were separated by electrophoresis and visualized using a Coomassie Blue Staining Kit (Beyotime, China, P0017A). Different bands between sense and antisense HOTAIRM1 were identified using mass spectrometry (Thermo Scientific, Bremen, Germany, 24600).

### RNA Immunoprecipitation Assay

An RNA immunoprecipitation (RIP) assay was used to determine whether HOTAIRM1 interacted with HOXA4/HSPA5 using the Magna RIP RNA-binding protein immunoprecipitation kit (Millipore, Billerica, MA, USA, 17–700). Cells were lysed with RIP buffer, and the cell extract was mixed with agarose beads carrying anti-HOXA4/HSPA5 or anti-lgG antibodies. Enrichment of the coprecipitated RNAs was detected by RT-qPCR.

### Statistical Analysis

Statistical data from each group were analyzed using GraphPad 6.0. Data are expressed as mean ± standard deviation (SD). Intergroup differences were assessed using Student’s *t*-test, and multivariate statistical analysis was performed using analysis of variance (ANOVA). Statistical significance was considered at *p* < 0.05.

## RESULTS

### HOTAIRM1 was Up-Regulated in the Presence of Ox-LDL

**Fig. 1:**
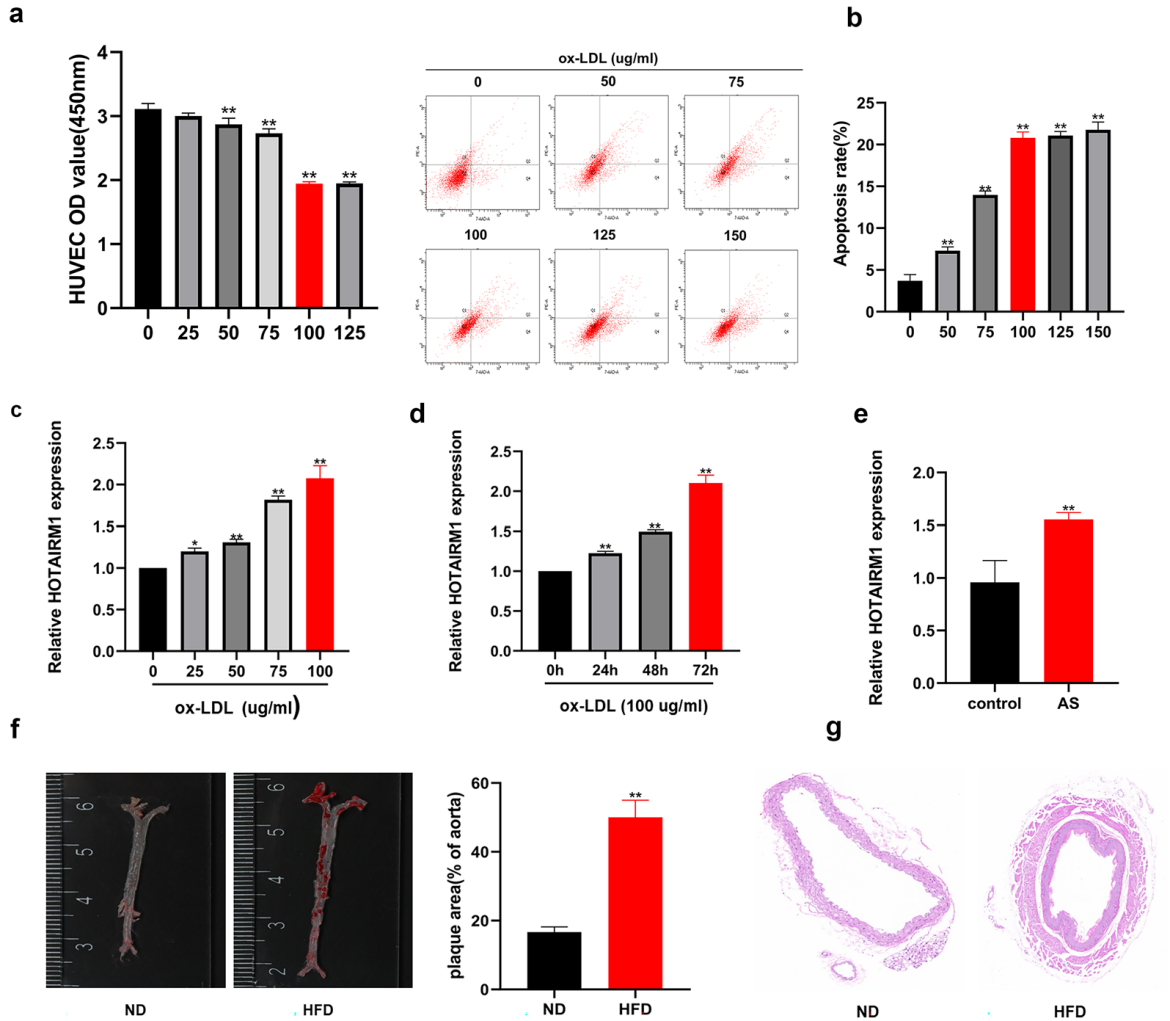
Establishment of ox-LDL treated HUVEC cell model and assessment of HOTAIRM1 expression. **a**, **b** HUVECs were treated with various concentrations of ox-LDL (0, 25, 50, 75, 100, 125 µg/ml) for 48 h, then cell viability was detected by CCK-8 assay (A), and cell apoptotic was tested by flow cytometry analysis (**b**). **c**, **d** The introduction of ox-LDL dose-dependently and time-dependently boosted the expression of HOTAIRM1 compared to the untreated group. **e** The expression of HOTAIRM1 in the aorta in HFD-fed ApoE − / − mice and in ND-fed ApoE − / − mice was measured by RT-qPCR. **f** The formation of aortic plaques in HFD-fed ApoE − / − mice and ND-fed ApoE − / − mice was detected by oil red O staining. **g** The formation of aortic plaques in HFD-fed ApoE − / − mice and ND-fed ApoE − / − mice was measured by H&E staining of the aortic arch. HUVECs, human umbilical vein endothelial cells; ox-LDL, oxidized low-density lipoprotein; ND, normal diet; HFD, high-fat diet. The data are presented as the mean ± SD; **p < 0.05, *p < 0.01. All experiments were independently repeated three times.

As a first step in elucidating the biological function of HOTAIRM1 in AS, ox-LDL was used to simulate a cell model of AS. To screen the appropriate ox-LDL concentration, the viability and apoptosis were measured in HUVECs stimulated with different ox-LDL concentrations (0, 25, 50, 75, 100, and 125 μg/mL). The results indicated that the introduction of ox-LDL impaired HUVEC viability and contributed to apoptosis compared to the untreated group (Fig. 1a, b). Ultimately, 100 μg/mL was selected for subsequent cell experiments. RT-qPCR was performed to examine whether the presence of ox-LDL affected HOTAIRM1 expression. Ox-LDL enhanced HOTAIRM1 expression in HUVECs in a dose- and time-dependent manner (Fig. 1c, d). In addition, Hotairm1 expression was up-regulated in the aorta of HFD-fed ApoE − / − mice (Fig. 1e). Oil red O and H&E staining represented that the area of arterial tissue damage in the ApoE − / − mice fed with high-fat-diet (HFD) was increased compared with that in the ApoE − / − mice fed with normal diet (ND) (Fig. 1f, g). These findings suggest that HOTAIRM1 is possibly a regulator in the biological regulation of HUVECs.

### HOTAIRM1 Depletion Blocked Proliferation and Expedited Apoptosis of HUVECs in Either the Absence or Presence of Ox-LDL

**Fig. 2:**
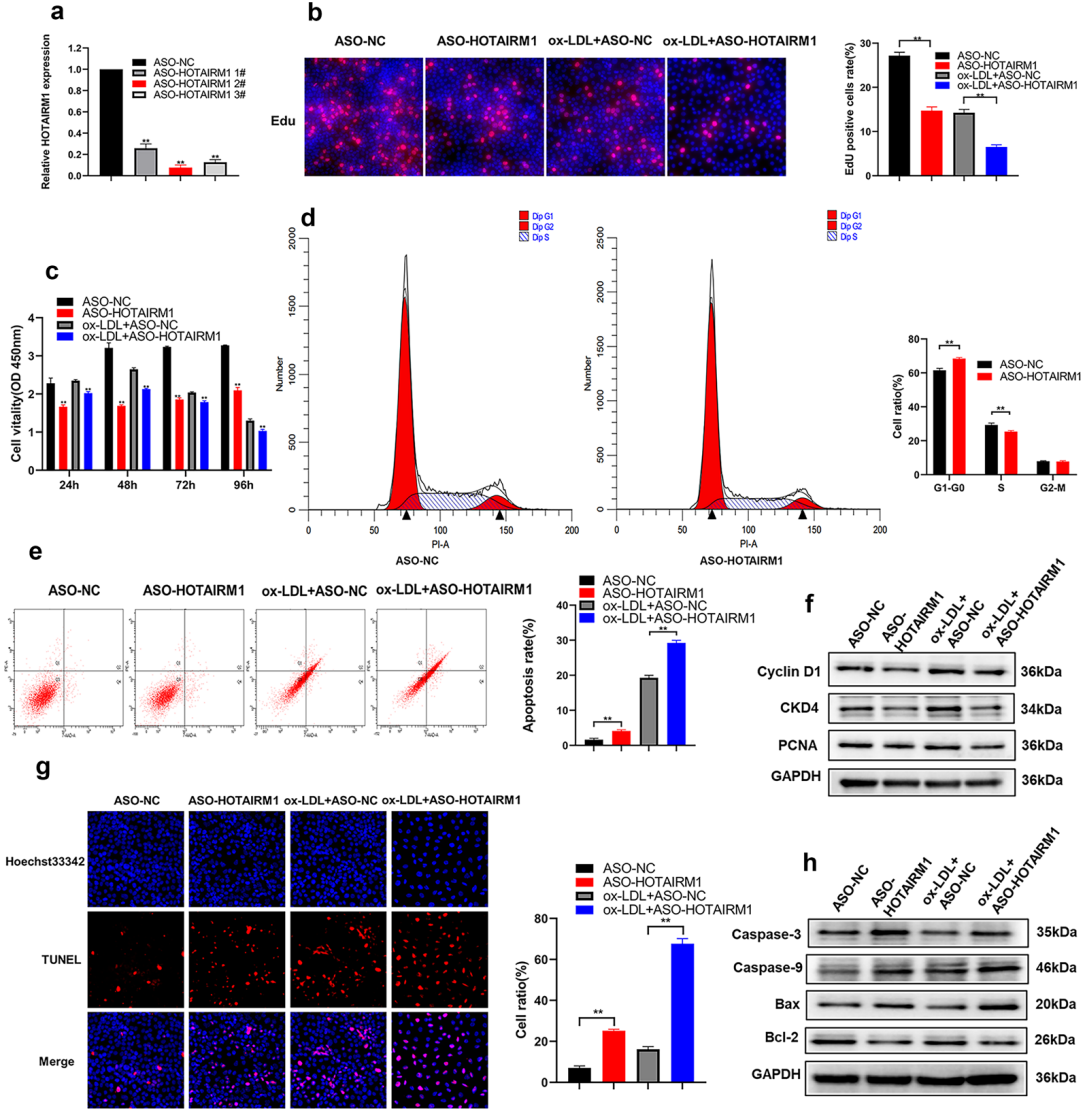
Knockdown of HOTAIRM1 suppressed cell proliferation and induced apoptosis in HUVECs in vitro. **c** The abundance of HOTAIRM1 in HUVECs transfected with ASO-NC, ASO-HOTAIRM1 1#, ASO-HOTAIRM1 2#, and ASO-HOTAIRM1 3# were examined by RT-qPCR. HUVECs were transfected with ASO-NC and ASO-HOTAIRM1 and then treated with or without 100 µg/ml ox-LDL for 24 h. **b** EdU staining assays were used to determine the proliferation of ASO-HOTAIRM1 transfected HUVECs. EdU-positive cells were counted and captured. Red represents EdU-positive cells and blue represents DAPI-stained nuclei. **c** CCK-8 assay was used to test the cell viability of HUVECs after transfection ASO of HOTAIRM1. **d** At 72 h after transfection, the cell cycle was analyzed by flow cytometry. The bar chart represents the percentage of cells in the G0/G1, S, or G2/M phase, as indicated. **e**, **g** The apoptosis of transfected HOTAIRM1 HUVECs was evaluated using Flow cytometry analysis assay (**e**) and TUNEL staining (**g**). **f** Western blot analysis of proliferation-related proteins in HUVECs transfected with ASO-HOTAIRM. GAPDH protein was used as an internal control. **h** The expression of apoptosis-related protein was examined by western blotting assay. The data are presented as the mean ± SD; **p < 0.05, *p < 0.01. All experiments were independently repeated three times.

Next, we sought to elucidate the biological role of HOTAIRM1 in HUVECs. Due to ox-LDL can induce HOTAIRM1 expression, ASOs that mediate HOTAIRM1 knockdown were screened; therefore, three individual ASOs were used to knock down their expression, and the silencing efficacy was evaluated by qPCR (Fig. 2a). In order to explore the effects of HOTAIRM1 on the proliferation and apoptosis of HUVEC, firstly, EdU and CCK-8 assays revealed that HOTAIRM1 knockdown remarkably reduced HUVEC proliferation irrespective of ox‐LDL treatment (Fig. 2b, c), and later, flow cytometry assay was performed to determine whether HOTAIRM1 depletion dampened cell proliferation by altering cell cycle progression. The results showed that the cell cycle was dramatically stalled at the G1-G0 phase in HOTAIRM1-silenced HUVECs (Fig. 2d), indicating that HOTAIRM1 deficiency impaired HUVEC proliferation, partly by regulating cell cycle progression. Furthermore, the silencing of HOTAIRM1 strikingly expedited HUVEC apoptosis (Fig. 2e, g). Finally, western blotting results revealed that the expression of cell cycle- and proliferation-related proteins, such as cyclin D1, CKD4, and PCNA, dramatically reduced upon HOTAIRM1 knockdown with a concurrent increase in apoptosis markers, including caspase-3, caspase-9, and Bax (Fig. 2F, H). Thus, HOTAIRM1 may play an important role in modulating HUVEC proliferation and apoptosis.

### HOTAIRM1 was Transcriptionally Activated by HOXA4

**Fig. 3:**
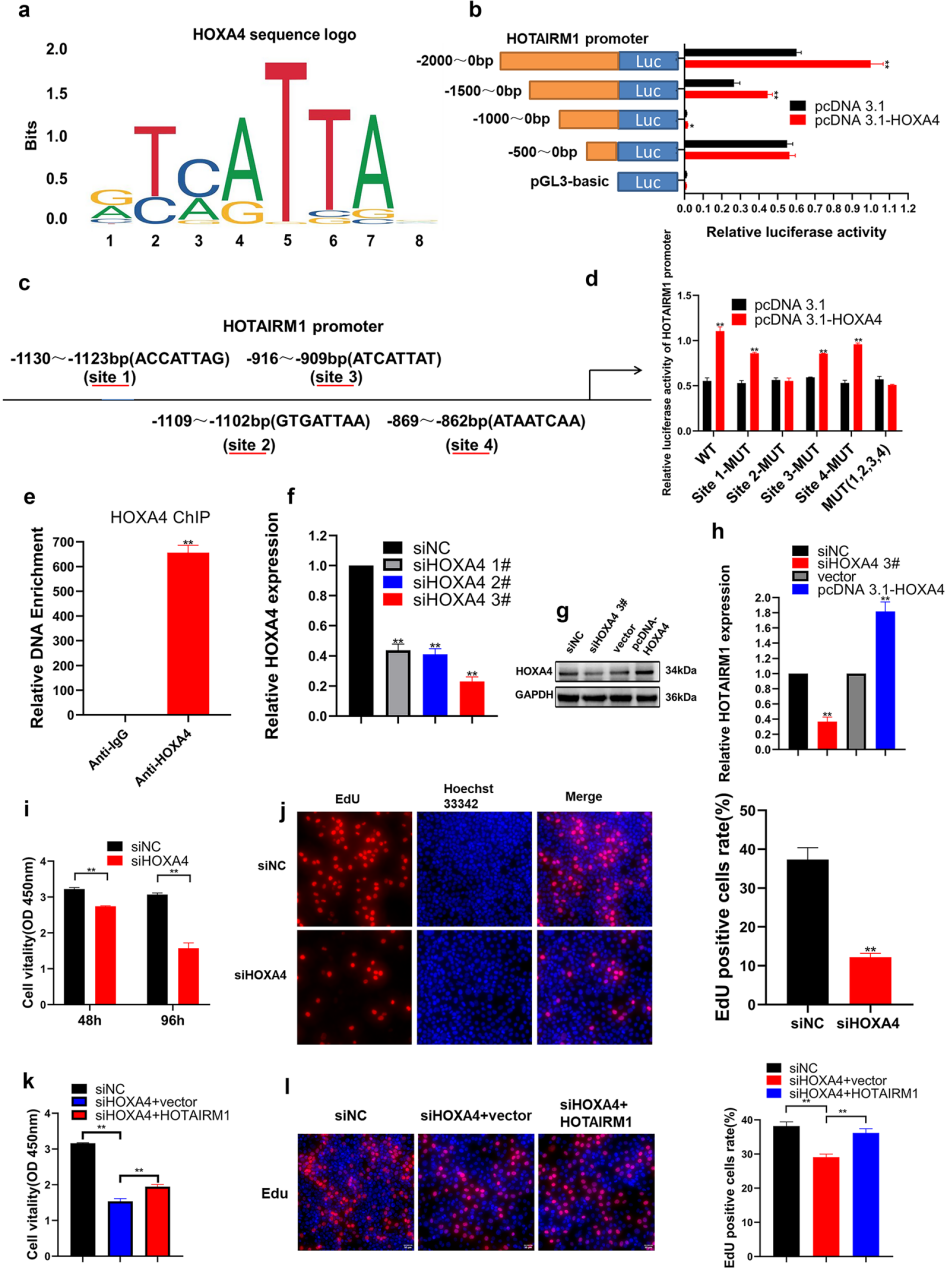
HOXA4 bound to the HOTAIRM1 promoter and directly activated HOTAIRM1 transcription. **a** The DNA binding motif for HOXA4 was predicted by JASPAR. **b** A dual-luciferase reporter assay was implemented by co-transfecting the full-length HOTAIRM1 promoter or deleted HOTAIRM1 fragment with HOXA4 plasmid or empty vectors in HUVECs. **c** Prediction of HOXA4 four binding sites in the HOTAIRM1 promoter region using JASPAR (http://jaspar.genereg.net/). **d** Luciferase reporter experiments were employed to detect the luciferase activity of indicated HOTAIRM1 promoter in HUVECs with or without HOXA4 overexpression. **e** ChIP assay showed that HOXA4 is significantly bound to the HOTAIRM1 promoter. IgG was used as a negative control. **f** HUVECs were transfected with siRNAs targeting HOXA4 or with control siRNAs and relative expression of HOXA4 was determined by RT-qPCR. **G** Western blotting analysis showing the protein levels of HOXA4 following the knockdown or overexpression of it in HUVECs. **h** HOXA4 overexpression increases, whereas HOXA4 knockdown decreases, the expression of HOTAIRM1. **i**, **j** Cell viability was detected by CCK-8 (**i**), and proliferation was detected by EdU (**J**) in HOXA4 silenced HUVECs. **k**, **l** CCK-8 (**l**) and EdU (**l**) assays exhibited that HOTAIRM1 overexpression could also reverse knockdown HOXA4-mediated proliferation suppression. The data are presented as the mean ± SD; ** p < 0.05, *p < 0.01. All experiments were independently repeated three times.

Mounting evidence suggests that the transcription of several lncRNAs can be regulated by transcription factors (TFs) via direct binding. To tease out potential upstream mediators exquisitely governing HOTAIRM1 in HUVECs, we conducted a motif analysis in the HOTAIRM1 promoter region using JASPAR (http://jaspar.binf.ku.dk/). The result implicated that HOXA4 might be a potential TF with probable binding motif “ATTA” (Fig. 3a), indicating that HOTAIRM1 may be modulated by HOXA4 at the transcriptional level. Because HOXA4 appeared to harbor numerous seemingly binding sites predicted by JASPAR in the HOTAIRM1 promoter region (Supplementary File 2), we constructed different HOTAIRM1 promoter truncations to identify the core promoter binding region. The HOTAIRM1 promoter region containing fragments of nucleotides − 2000 to 0 bp, − 1500 to 0 bp, − 1000 to 0 bp, and − 500 to 0 bp were cloned into a PGL3 luciferase reporter construct. When the reporter was co-transfected with a HOXA4 overexpression construct into HUVECs, it displayed remarkable activation by HOXA4 within the promoter region of HOTAIRM1 ranging from − 2000 to 0 bp, − 1500 to 0 bp, and − 1000 to 0 bp. Furthermore, compared to the control group, multiple luciferase activities were enhanced in the HOTAIRM1 promoter regions. Luciferase activity from − 2000 to 0 bp was akin to that from − 1500 to 0 bp, while luciferase activity from − 1500 to 0 bp was higher than that from − 500 to 0 bp. However, there was no influence on the promoter activity of HOTAIRM1 in the region from − 500 to 0 bp (Fig. 3b). A thorough analysis of the aforementioned results showed that HOXA4 promotes HOTAIRM1 transcription. Of note, the core binding region located between − 1500 and − 500 bp was defined, and the JASPAR website again predicted four possible binding sites for HOXA4 in this region, namely, binding site 1 located between − 1130 and − 1123 bp (ACCATTAG), site 2 between − 1109 and − 1102 bp (GTGATTAA), site 3 between − 916 and − 909 bp (ATCATTAT), and site 4 between − 869 and − 862 bp (ATAATCAA) of the HOTAIRM1 promoter region (Fig. 3c). To identify the specific binding site of HOXA4 responsible for its binding to HOTAIRM1, a series of small-scale deletion mutants were generated. As shown in Fig. 3d, HOXA4 overexpression led to a substantial increase in the luciferase activity of HOTAIRM1 promoter with wild-type or sites 1/3/4 mutations in HUVECs, with barely altered luciferase activity of HOTAIRM1 promoter with site 2 or all four sites mutations, thereby revealing that the site 2 with − 1109 to − 1102 bp was important for HOXA4-mediated HOTAIRM1 activation. ChIP-qPCR assay was used to further examine the role of HOXA4 in the HOTAIRM1 promoter region. Interestingly, the occupation of HOXA4 in the HOTAIRM1 promoter region was substantially increased compared to that in the IgG control (Fig. 3e). Collectively, the investigation of transcriptional regulation revealed that HOXA4 directly binds to the HOTAIRM1 promoter and facilitates its transcription.

The regulatory role of HOXA4 on HOTAIRM1 has not been addressed thus far. The siRNAs specifically targeting HOXA4 were generated, and silencing efficiency was evaluated by qPCR and western blotting (Fig. 3f, g). Indeed, knocking down or overexpressing HOXA4 led to decreased or increased HOTAIRM1 expression, respectively (Fig. 3h). Functionally, CCK-8 and EdU assays showed that HOXA4 knockdown dampened the proliferation of HUVECs (Fig. 3i, j). Whether HOTAIRM1 is involved in the contribution of HOXA4 to cell proliferation regulation remains unknown. Rescue experiments showed that HOTAIRM1 overexpression partially counteracted the HOXA4 depletion-mediated proliferation impairment (Fig. 3k, l). Cumulatively, the effect may account for the fact that HOXA4 functions, at least in part, by being contingent upon HOTAIRM1 and indicates that HOXA4 can bind and activate the HOTAIRM1 promoter.

### Positive Feedback Signaling Between HOTAIRM1 and HOXA4 in HUVECs

**Fig. 4:**
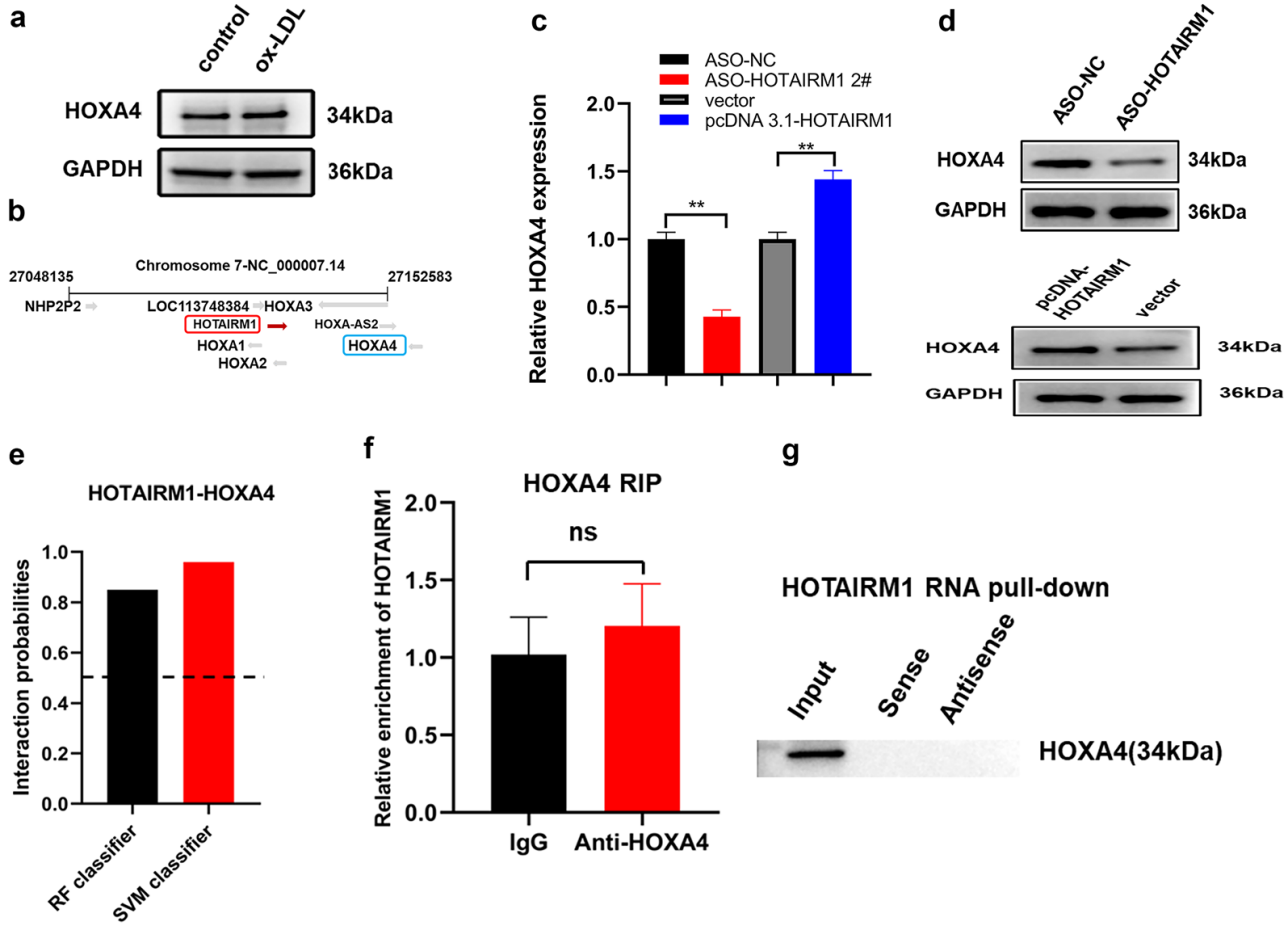
HOTAIRM1 regulated the expression of HOXA4 in a non-RBP manner. **a** Protein level of HOXA4 in HUVECs treated with ox-LDL. **b** HOTAIRM1 is located next to the HOXA4 gene on human chromosome 7. HOTAIRM1 is encoded by the ( −) DNA strand, while HOXA4 is coded by the ( +) DNA strand. **c**, **d** HOTAIRM1 regulates HOXA4 at the transcriptional level. Knocking down or overexpressing HOTAIRM1, respectively, downregulates or upregulates HOXA4. **e** The interaction probability of HOTAIRM1 with HOXA4 was predicted by the RNA–Protein interaction prediction (RPISeq) website. Predictions with probabilities > 0.5 were considered “positive,” indicating that RNA and protein are likely to interact. **f** RIP assay showed the enrichment of HOTAIRM1 in anti-HOXA4 groups, and lgG was used as a negative control. **g** RNA pull-down experiment was conducted to detect the enrichment of HOXA4 in HOTAIRM1 groups. **p < 0.05, *p < 0.01; ns not significant. All experiments were independently repeated three times.

The introduction of ox-LDL led to a dramatic increase in the protein level of HOXA4 in HUVECs (Fig. 4a). To gain insight into how HOTAIRM1 affects cell proliferation, we first examined the localization of HOTAIRM1 through NCBI (https://www.ncbi.nlm.nih.gov/). HOTAIRM1 and HOXA4 are located adjacent to the same chromosome and in opposite directions of transcription (Fig. 4b). Existing data have demonstrated that lncRNAs can function in cis to regulate the expression of adjacent protein-coding genes [31], indicating that HOTAIRM1 may modulate HOXA4 expression, which has yet to be imprinted in HUVECs. HOTAIRM1 knockdown substantially diminished, whereas its overexpression markedly augmented HOXA4 expression at the mRNA and protein levels (Fig. 4c, d). To further elucidate how HOTAIRM1 influences HOXA4 expression, the possible interaction between HOTAIRM1 and HOXA4 was assessed using bioinformatics analyses. Using the RNA–protein interaction prediction (RPISeq) website (http://pridb.gdcb.iastate.edu/RPISeq/), HOXA4 was shown to interact with HOTAIRM1 using the random forest (RF) classifier (= 0.90) and support vector machine (SVM) classifier (= 0.96) (Fig. 4e), suggesting that HOTAIRM1 could interact with HOXA4. Inspired by these observations, follow-up RIP, RNA pull-down, and western blotting experiments were performed to determine whether HOTAIRM1 could interact with HOXA4 in an RBP manner. Unfortunately, the experimental results described here yielded no evidence of the corresponding RBP patterns (Fig. 4f, g). Overall, these discoveries, coupled with our existing knowledge presented above on the transcription promotion of HOTAIRM1 initiated by HOXA4, support the notion that HOTAIRM1 tuned the expression of HOXA4 in a non-RBP manner. Of course, this raises a major intriguing problem in the HOTAIRM1-mediated regulatory axis, which is a candidate for downstream HOTAIRM1-associated protein partners.

### Identification of HSPA5 as a Binding Partner for HOTAIRM1 in HUVECs

**Fig. 5:**
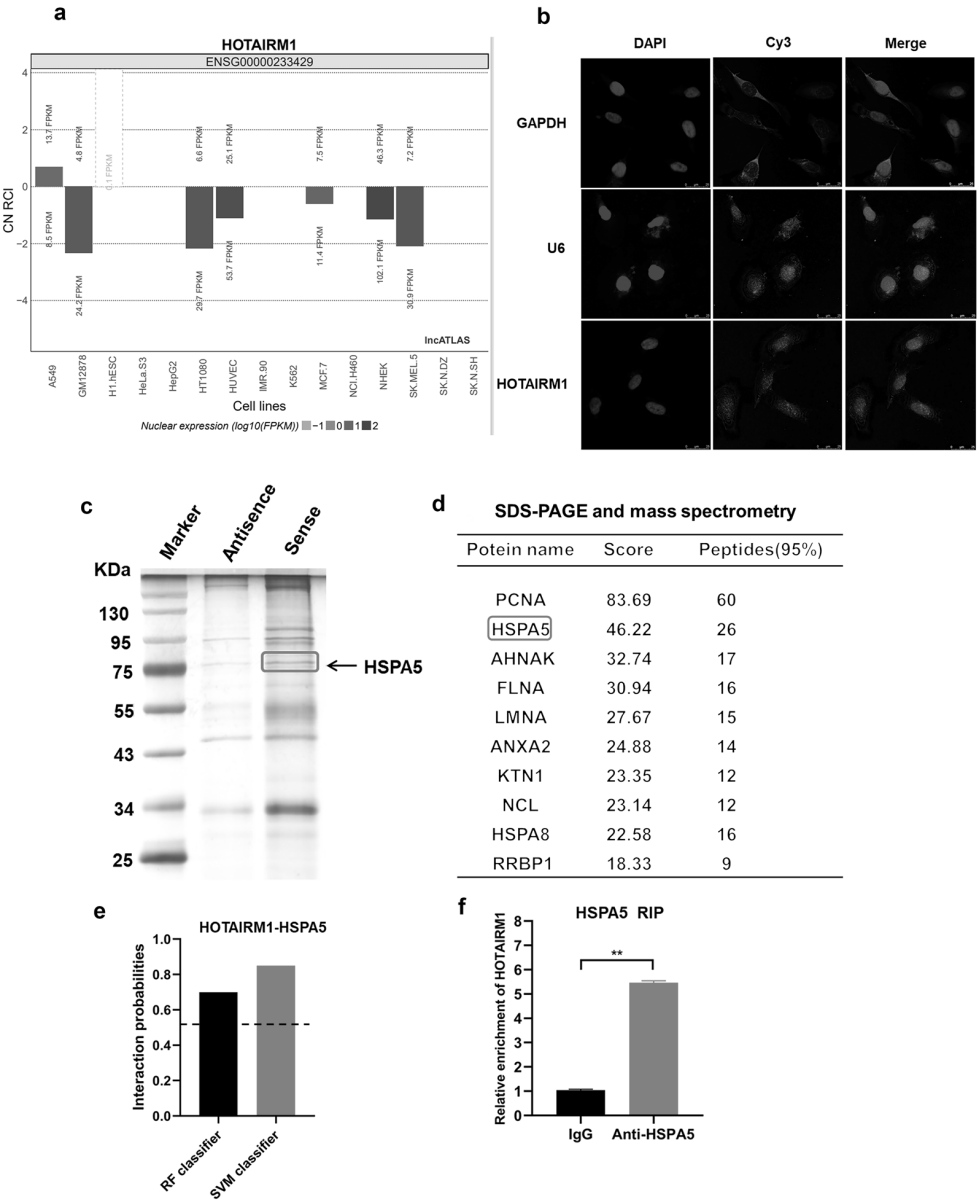
HOTAIRM1 interacted with HSPA5. **a** The expression location of HOTAIRM1 was predicted using the lncATLAS (http://lncatlas.crg.eu/), with the 0 line below representing HOTAIRM1 expression in the nucleus and the 0 line above representing HOTAIRM1 expression in the cytoplasm. **b** The subcellular localization of HOTAIRM1 visualized by FISH, the fluorescence image showed the results of FISH assay, DAPI-stained nucleus: blue; Cy3 at the 5′ end of the probe (HOTAIRM1 or U6): red; the merged image represents the overlap of DAPI and HOTAIRM1. Scale bar = 50 µm, 200 × . **c**, **d** RNA pull-down, silver staining, and mass spectrometry analysis were performed to identify specific proteins that bind to HOTAIRM1. Biotin-labeled HOTAIRM1 was incubated with lysates from HUVECs to pull down the HOTAIRM1-associated proteins, followed by silver staining. A specific fragment (indicated by an arrow), pulled down by HOTAIRM1 (**c**), but not by antisense RNA, was subjected to mass spectrometry, the red square indicates the approximate position of HSPA5 (75 kDa). The top ten candidate proteins are listed (**d**). **e** The prediction of the interaction probabilities of HOTAIRM1 with RNA-binding protein HSPA5 by Bioinformatics (http://pridb.gdcb.iastate.edu/RPISeq/). Predictions with probabilities > 0.5 were considered “positive,” indicating that the corresponding RNA and protein are likely to interact. **f** RIP assays were performed to verify HOTAIRM1 binding to HSPA5 using an anti-HSPA5 antibody; lgG was used as a negative control. RIP, RNA immunoprecipitation. **p < 0.05, *p < 0.01. All experiments were independently repeated three times.

To delineate the RNA-binding protein landscape in the HOTAIRM1-mediated signaling network, we aimed to identify HOTAIRM1-associated proteins responsible for modulating cell proliferation. The subcellular localization of lncRNAs determines their modes of action [32]. Hence, the subcellular location of HOTAIRM1 was predicted using lncATLAS (https://lncatlas.crg.eu/), and the results indicated that it was preferentially localized in the nuclei of HUVECs (Fig. 5a). To unequivocally visualize HOTAIRM1 distribution as described by the RNA-FISH assay, it was also principally located in the nucleus of HUVECs (Fig. 5b), which enabled HOTAIRM1 to play an important role at the transcriptional level. Overwhelming evidence has conferred multiple regulatory functions to lncRNAs via interactions with diverse RNA-binding protein partners in the nucleus. Toward this end, HOTAIRM1-interacted proteins were identified and characterized by RNA pull-down assay, followed by mass spectrometry in HUVECs (Fig. 5c). Consequently, 233 proteins associated with HOTAIRM1 were identified using mass spectrometry (Supplementary File 3), and HSPA5 was identified as a prominent HOTAIRM1-binding protein among the top ten hit candidate proteins (Fig. 5d).

To further confirm the HOTAIRM1-HSPA5 interaction, the RNA–protein interaction prediction (RPISeq) website (http://pridb.gdcb.iastate.edu/RPISeq/) was used to predict whether HOTAIRM1 could bind to the HSPA5 protein. The scores for the random forest (RF) classifier and support vector machine (SVM) classifier are 0.7 and 0.85 (both over 0.5) (Fig. 5e), respectively, raising the likelihood that HOTAIRM1 could interact with HSPA5. A follow-up RIP experiment was carried out for the RNA-HSPA5 complex utilizing the HSPA5 antibody to corroborate this speculation. Notably, upon comparison with the IgG-control group, HSPA5 caused a robust HOTAIRM1 enrichment in HUVECs (Fig. 5f). Overall, these data indicate that HOTAIRM1 physically interacts with HSPA5 in HUVECs in an RBP manner.

### HOTAIRM1 Regulated HSPA5 Expression in a Transcription-Dependent Manner and its Effect on Proliferation was HSPA5 Partially Dependent

**Fig. 6:**
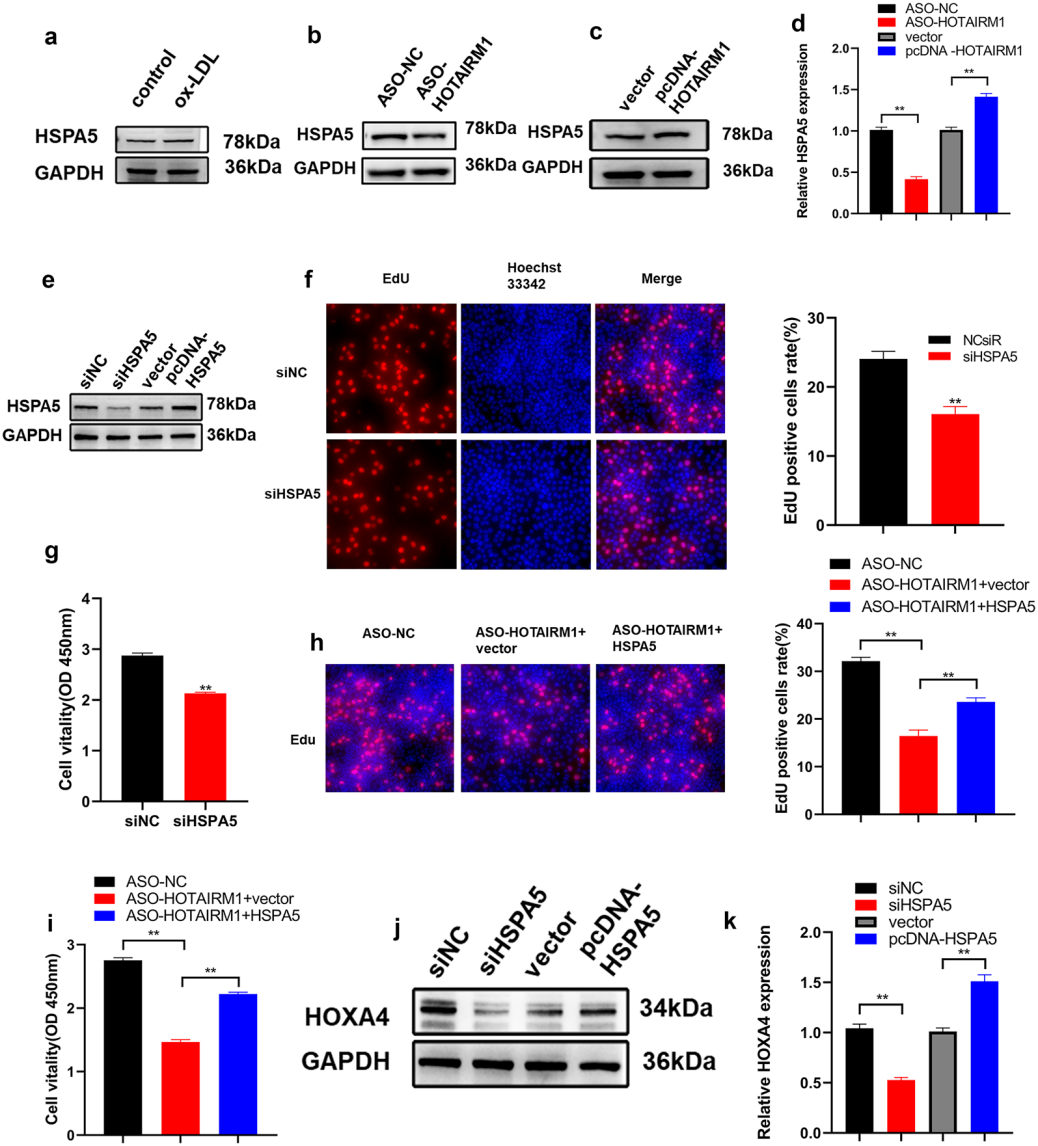
HOTAIRM1 regulated the expression of HSPA5 at the transcriptional level. **a** Protein level of HSPA5 in HUVECs with ox-LDL inducing. **b**–**d** HOTAIRM1 knockdown decreases, whereas HOTAIRM1 overexpression increases, HSPA5 expression at mRNA and protein levels. **e** Western blotting analysis showing the protein levels of HSPA5 following the knockdown or overexpression of it in HUVECs. **f**, **g** EdU and CCK-8 assays showed that the knockdown of HSPA5 could suppress cell proliferation. **h**, **i** EdU and CCK-8 assays showed that HSPA5 overexpression could reverse knockdown HOTAIRM1-mediated proliferation suppression. **j**, **k** HSPA5 knockdown repressed, whereas HSPA5 overexpression enhanced, HOXA4 expression at mRNA and protein levels. Shown are three independent experiments. **p < 0.05, *p < 0.01. All experiments were independently repeated three times.

Based on these results, we further investigated whether HOTAIRM1 could regulate HSPA5 expression in HUVECs and found that the protein level of HSPA5 was markedly up-regulated in HUVECs (Fig. 6a), which was in accordance with HOTAIRM1 expression in the presence of ox-LDL. The gain-and-loss-of-function experiments were performed to elucidate the regulatory role of HOTAIRM1 in HSPA5 expression. HOTAIRM1 knockdown mitigated this effect; however, its overexpression increased HSPA5 protein expression (Fig. 6b, c). Down-regulation or up-regulation of HOTAIRM1 remarkably decreased or increased HSPA5 expression at the mRNA level, respectively (Fig. 6d), indicating that HOTAIRM1 modulates the expression of HSPA5 in a transcription-dependent manner. To determine whether HSPA5 is responsible for the role of HOTAIRM1 in HUVEC proliferation, siRNA and overexpression plasmids specifically targeting HSPA5 were utilized to evaluate the protein expression level in HUVECs (Fig. 6e). EdU and CCK-8 assays demonstrated that HSPA5 knockdown markedly retarded HUVEC proliferation (Fig. 6f, g). We then transfected HOTAIRM1-silenced HUVECs with an HSPA5-overexpressing plasmid or empty vector and found that HSPA5 overexpression partly countered the effect of HOTAIRM1 depletion on HUVEC proliferation suppression (Fig. 6h, i), thereby showing that HOTAIRM1-mediated proliferation modulation was dependent on HSPA5. Cell fate determination was tightly orchestrated by a series of molecular signals to unveil the regulatory network of the HOXA4-HOTAIRM1-HSPA5 axis, and the effect of HSPA5 on HOXA4 expression was monitored. HSPA5 knockdown blunted, whereas its overexpression potentiated HOXA4 expression at the mRNA and protein levels in HUVECs (Fig. 6j, k).

## DISCUSSION

The discovery of lncRNAs has opened up a new chapter and revolutionized the molecular and cellular biology fields. In recent years, studies on lncRNA biology have shown that they represent a diverse and prevalent group of RNAs and have emerged as appealing orchestrators at the forefront of AS and associated cardiovascular events. AS is a ubiquitous phenomenon that underlies the initiation and progression of several cardiovascular disorders. Aberrant endothelial cell proliferation is closely related to various cardiovascular diseases, including AS; however, whether lncRNA HOTAIRM1 is also involved in this process remains unclear. Ox-LDL has long been associated with AS [33, 34]. For this purpose, the detailed molecular underpinning of HOTAIRM1 in the ox-LDL milieu is important for a deeper understanding of AS. A HUVEC model exposed to ox-LDL was initially used to investigate AS. As such, it is unclear whether HOTAIRM1 is responsive to ox-LDL or whether HOTAIRM1 yields profound insights into its function in response to ox-LDL stimuli in HUVECs. To clarify these issues, we quantified the expression of HOTAIRM1 and demonstrated its effects on the related phenotypes in the absence and presence of ox‐LDL. In this study, a blueprint of functional and mechanistic associations was shaped; ox-LDL-driven HOTAIRM1 was activated by HOXA4 in HUVECs via direct association with its promoter. A barrage of subsequent functional analyses of HOTAIRM1 revealed a reduction of its knockdown on proliferation and noteworthy promotion of apoptosis in HUVECs with or without ox-LDL, suggesting that HOTAIRM1 may be an extraordinarily vital factor in the regulation of proliferation in HUVECs. These findings may open the intriguing perspective of HOTAIRM1 as a plausible therapeutic target for stemming AS.

Little is known about the upstream signals responsible for triggering HOTAIRM1 expression, although HOTAIRM1 is involved in the biological behavior of cells by modulating downstream target genes. Uncovering why HOTAIRM1 is highly expressed in ox-LDL-treated HUVECs is crucial for probing the relevant regulatory circuits. Therefore, we describe HOXA4 as a novel upstream transcription factor that activates HOTAIRM1. HOXA4, a member of the HOX gene family, functions as a transcription factor that regulates the expression of downstream genes to build segments or structures within the body [35]. Mechanistically, the mode of action is mainly via transcriptional activation or repression of target genes [36]. Previous reports have shown that HOXA4 acts as a tumor suppressor in various cancers [37]. A recent report uncovered that HOXA4 affected lung cancer progression by inhibiting the Wnt-b-catenin signaling through GSK3B up-regulation [38]. For the implication of HOXA4 in tumorigenesis, multiple studies have been conducted to elaborate the transcription landscape. For the most part, these findings largely underscored protein-coding genes, delving deeply into their regulatory network, especially at the lncRNA layer, which will be beneficial for unraveling relevant regulatory mechanisms. More specifically, the mechanism by which HOXA4 modulates cell proliferation by regulating the transcription of lncRNAs remains unknown. Therefore, HOXA4 occupies the promoter region of HOTAIRM1 as a transcriptional activator, at least to some extent, which may be partially responsible for HOTAIRM1 up-regulation in HUVECs exposed to ox-LDL. Foremost among these was a key binding site with the base sequence “GTGATTAA” as the HOXA4 response element in the HOTAIRM1 promoter that mediated its transcription activation. Structurally, HOXA4, one of the homeobox family genes, comprises two exons and one intron. Its function depends on an evolutionarily conserved 60 amino-acid homeodomains critical for DNA binding at specifically identified sites covering only four base-pair sequences (TAAT/ATTA/TTAT/ATAA), indicating low functional specificity [39, 40]. The mainly identified binding site base sequence herein is “GTGATTAA,” including the consensus sequence “ATTA,” which is consistent with previous investigation. Functionally, in vitro HOTAIRM1 impairment blunted HUVEC proliferation. Inhibition of proliferation elicited by HOXA4 depletion was partially abrogated by HOTAIRM1 overexpression in HUVECs. In summary, upon HOXA4 reduction, the restored proliferation rates of HUVECs could be attributed to enhanced HOTAIRM1 levels, indicating that HOTAIRM1, a downstream target gene of HOXA4, performs its function through its involvement in HOXA4-mediated proliferation modulation.

A plethora of lncRNAs has been proposed to regulate gene expression in a cis- or trans-manner [41], as exemplified by DEANR1-mediated DE differentiation by regulating its adjoining gene FOXA2 [42]. More than 60% of the endoderm-expressed lncRNAs exhibit coordinated expression changes with nearby protein-coding genes [43]. Thus, we investigated whether HOTAIRM1 facilitated HUVEC proliferation by regulating HOXA4 expression. As mentioned previously, an intriguing discovery was the interplay between HOTAIRM1 and its adjacent protein-coding gene *HOXA4* in HUVECs. Scilicet, HOXA4-activated HOTAIRM1, in turn, regulates HOXA4 expression, eventually forming a positive feedback signal to ensure HOTAIRM1 expression. Therefore, we further investigated the possible interaction between HOTAIRM1 and HOXA4 protein in a RBP manner. Notably, the interrogation was at odds with aforehand prediction work, a failure to observe the corresponding regulation between HOTAIRM1 and HOXA4. Accordingly, alternative or additional patterns of HOTAIRM1-mediated HOXA4 regulation cannot be excluded. A previous report showed that HOTAIRM1 recruits EZH2 and SUZ12 to the promoter of the target gene HOXA1, leading to the trimethylation of histone H3K27 and epigenetic silencing of HOXA1 [44]. Therefore, we postulated that HOTAIRM1 might regulate the expression through HOXA4 epigenetic regulation. Besides the probable epigenetic surveillance mechanism, other regulatory mechanisms may exist that are yet to be documented. Although we have thus far failed to unveil convincing evidence, further substantiation may potentially unmask a sparkling aspect of the regulatory networks afforded by HOTAIRM1 at the epigenetic level.

As previously shown, lncRNAs regulate gene expression at the pre-transcriptional, transcriptional, and post-transcriptional levels [45]. lncRNA modulation of cellular processes hinges partly on cellular localization; nuclear lncRNAs are abundant for functionality implicated in chromatin interactions, transcriptional regulation, and RNA processing, whereas cytoplasmic lncRNAs can modulate mRNA stability or translation and affect cellular signaling cascades [46]. The interaction of lncRNAs with functional partners, particularly proteins, remains largely unknown and has recently become an emerging research hotspot. Much effort is being devoted to identifying these interacting partners as a strategy for gaining insight into the molecular mechanisms. lncRNA regulation of genes is in the spotlight because of their enrichment in the nucleus, where they perform various cellular biological processes, often through their ability to directly interact with proteins [47–49]. Herein, an initial analysis of the subcellular localization of HOTAIRM1 using online lncATLAS prediction and fluorescent in situ hybridization showed an overwhelming enrichment in the nucleus of HUVECs, suggesting that HOTAIRM1 may regulate gene expression at the transcriptional level. RNA pull-down and liquid chromatography-mass spectrometry (LC–MS) were used to screen HOTAIRM1-associated proteins. These data allowed us to identify HSPA5 as an important binding partner of HOTAIRM1 in HUVECs. RIP assay revealed the binding of HOTAIRM1 to HSPA5. However, an additional aspect related to this binding has emerged: which region of HOTAIRM1 interacts with HSPA5. Evidence of specific binding region is currently lacking, and follow-up deletion mapping experiments will be undertaken to dissect the structural determinant elements for such reciprocal interactions in the future. Beyond mutual interactions, HOTAIRM1 down-regulation or up-regulation also resulted in decreased or increased HSPA5 levels in HUVECs. Similar to HOTAIRM1 expression, HSPA5 was also up-regulated in HUVECs exposed to ox-LDL. Functionally, the silencing of HSPA5 led to impaired HUVEC proliferation, comparable to that acquired by HOTAIRM1 knockdown.

We articulate whether HOTAIRM1-mediated proliferation is required for HSPA5 involvement. HSPA5 overexpression can largely lead to the reverse of the proliferation phenotype observed upon HOTAIRM1’s loss of function, suggesting that HSPA5 is a major functional target of HOTAIRM1. Collectively, HOTAIRM1, a crucial factor in HUVEC proliferation regulation, functions by directly interacting with and modulating HSPA5. Our discovery of such interactions expanded the view of the role of HOTAIRM1, including its functional involvement as both a lncRNA itself and a player that exerts its effect through RNA–protein interaction in HUVECs.

**Fig. 7:**
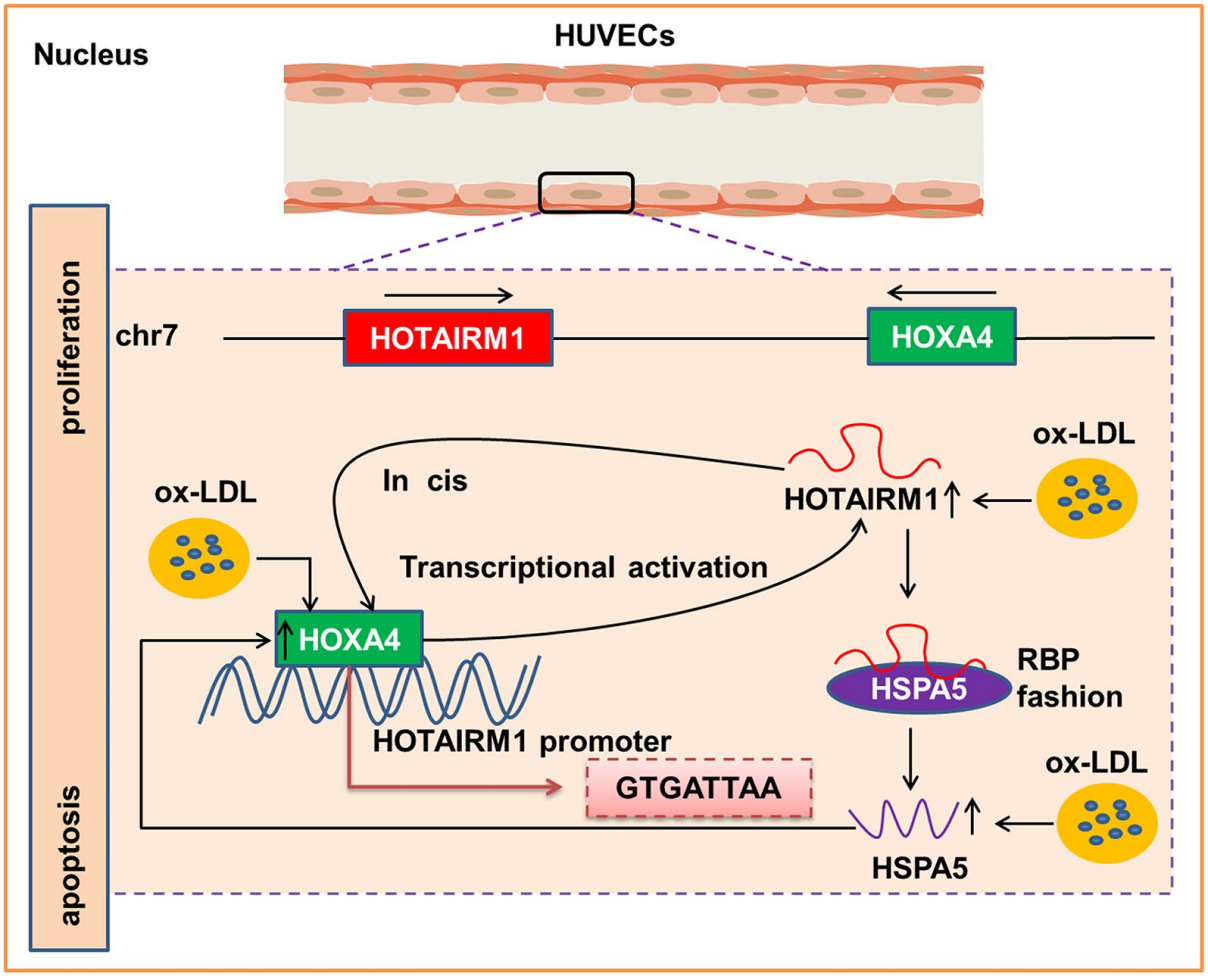
A schematic diagram for the mechanisms that illustrated the upstream activator and downstream effector of HOTAIRM1 in HUVECs. The genomic locations of HOTAIRM1 and HOXA4 genes are exhibited on the top line and the arrows show transcription direction. The binding of HOXA4 to the HOTAIRM1 promoter region initiated its transcription, a pivotal binding site with the base sequence “GTGATTAA” as the HOXA4 response element in the HOTAIRM1 promoter was determined. Meanwhile, HOTAIRM1 in turn regulated the expression of HOXA4 in cis forming a positive feedback circuit with HOXA4. Upon HOTAIRM1 activation, interacting of transcribed HOTAIRM1 with HSPA5 in RBP fashion facilitated the expression of HSPA5, thus contributing to HUVECs proliferation.

Ultimately, there were several emphases described herein that were worth reiterating: HOTAIRM1, a functional lncRNA identified in this report, was up-regulated in the presence of ox-LDL and was required for HUVEC proliferation. We emphasized that HOTAIRM1 in HUVECs, transcriptionally activated by HOXA4, coupled with HOTAIRM regulation exhibited a positive feedback signaling pattern. Moreover, HOTAIRM1 played its action in HUVEC proliferation modulation, in part, by interacting with and reclining on HSPA5. In conclusion, our findings identified HOTAIRM1 as an orchestrator of HUVEC proliferation and revealed a heretofore uncharacterized regulatory mechanism by which HUVEC proliferation is tightly governed by HOXA4-HOTAIRM1-HSPA5-driven axis. A summary of these mechanisms is presented in Fig. 7. As shown previously and substantiated herein, thorough mechanistic studies of the fundamental actions of HOTAIRM1 and the identification of its associated protein partners are critical for the exploitation and fulfillment of appealing therapeutic targets for AS. This study may pave the way for addressing the momentous question in HOTAIRM1-mediated regulation of HUVEC proliferation and AS. Although very promising, this study still has limitation exists because it principally focused on in vitro culture experiments with little functional data derived from animal evidence. Consequently, visualization of the therapeutic effects of targeting HOTAIRM1 or pertinent molecules could be performed using an animal model of AS, which is certainly subject to in-depth investigation. Thus, trailblazing research remains to be conducted in the field in the future. Although unprecedented advances have been made, many questions and challenges associated with HOTAIRM1 remain. Further thorough investigations may lead to ground-breaking results regarding AS development.

### Supplementary Information

Below is the link to the electronic supplementary material.

Supplementary file1 (DOCX 16 KB)

Supplementary file2 (TIF 500 KB)

Supplementary file3 (XLSX 39 KB)

Supplementary file4 (RAR 1178 KB)

Supplementary file5 (TIF 361 KB)

## Data Availability

The data that support the findings of this study are available on request from the corresponding author, upon reasonable request.

## Abbreviations

ASCVD: Atherosclerotic cardiovascular disease
AS: Atherosclerosis
ASOs: Antisense oligonucleotides
CCK-8: Cell Counting Kit-8
EdU: 5-Ethynyl-2′-deoxyuridine
GSK3B: Glycogen synthase kinase 3 beta
HOTAIRM1: HOXA transcript antisense RNA myeloid-specific 1
HUVECs: Human umbilical vein endothelial cells
LncRNAs: Long non-coding RNAs
LC-MS: Liquid chromatography-mass spectrometry
NCBI: National Center for Biotechnology Information
OX-LDL: Oxidized low-density lipoprotein
PCNA: Proliferating cell nuclear antigen
RBP: RNA-binding protein
siRNAs: Small interfering RNAs
TUNEL: TdT-mediated biotin-16-dUTP nick-end labeling
RIP: RNA immunoprecipitation
VECs: Vascular endothelial cells
