# Supplementary material for: lncRNA HOTAIRM1 Activated by HOXA4 Drives HUVEC Proliferation Through Direct Interaction with Protein Partner HSPA5
Source: Inflammation. 2023 Oct 29;47(1):421–37. doi: 10.1007/s10753-023-01919-x (PMC10798933; doi:10.1007/s10753-023-01919-x)
Supplement: Supplementary file 1 — Supplementary file1 (DOCX 16 KB) [file 10753_2023_1919_MOESM1_ESM.docx]

| Primer | Sequence (5’-3’) |
| --- | --- |
| HOTAIRM1-F | TGTTTTGCCTGAACCCATCAA |
| HOTAIRM1-R | CCCCCAACTCCAGGAATGA |
| HOXA4-F | TCAGCGCCGTTAACCCCAGTTA |
| HOXA4-R | TGACCTGGCGCTCAGACAAA |
| HSPA5-F | CAGTTGTTACTGTACCAGCCTA |
| HSPA5-R | CATTTAGGCCAGCAATAGTTCC |
| β-actin-F | TCATCTTCTCGCGGTTGGC |
| β-actin-R | TGGCACCACACCTTCTACAATG |
| Hotairm1-F (mice） | TCCCCACCCAGCCGAGAGAACTGAA |
| Hotairm1-R (mice) | TTCCTTCCCCTCCCCCCATTTTC |

**Table S1**. List of primers.
