# Supplementary figures and images for: lncRNA HOTAIRM1 Activated by HOXA4 Drives HUVEC Proliferation Through Direct Interaction with Protein Partner HSPA5
Source: Inflammation. 2023 Oct 29;47(1):421–37. doi: 10.1007/s10753-023-01919-x (PMC10798933; doi:10.1007/s10753-023-01919-x)

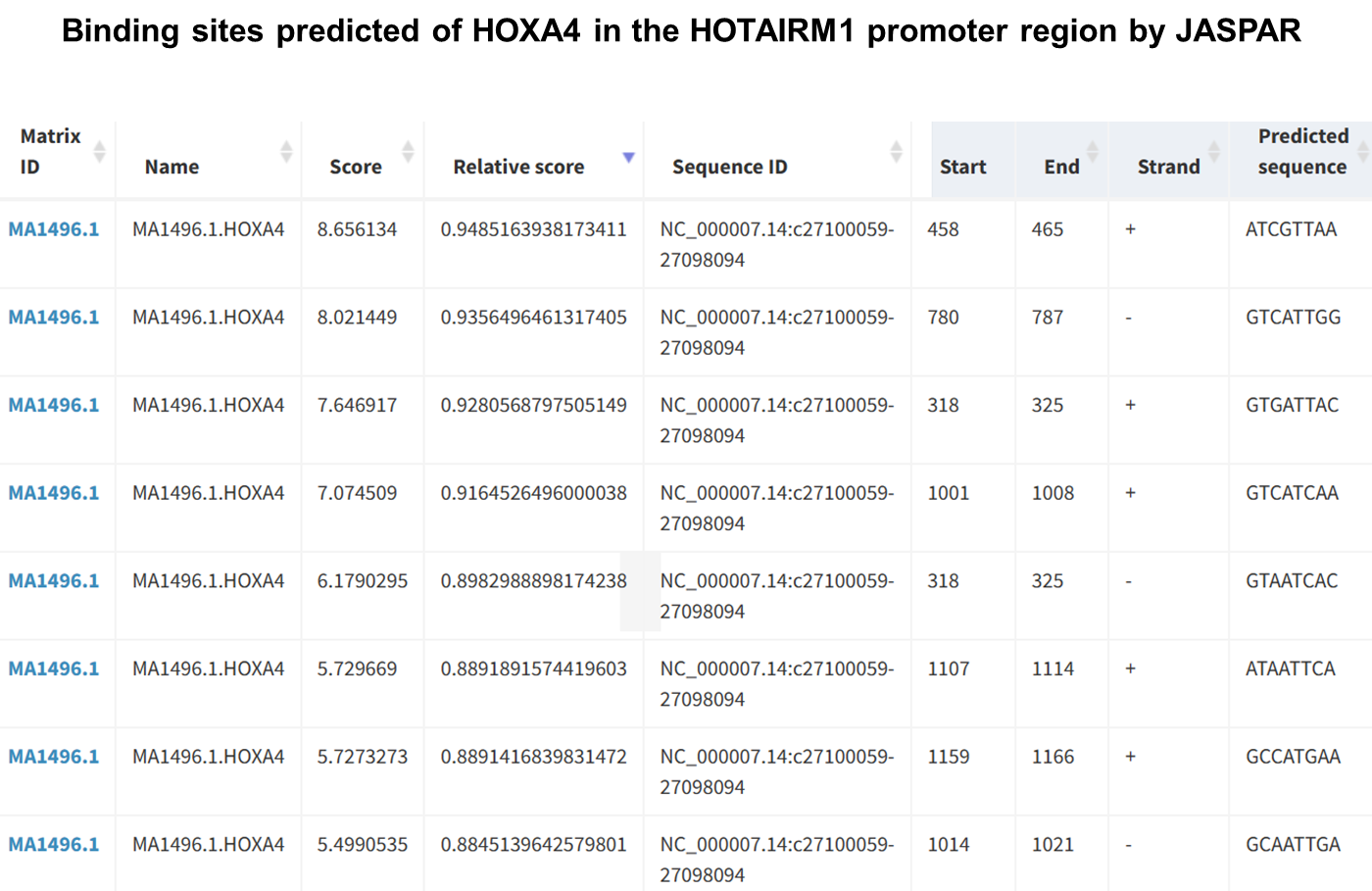

Supplement: Supplementary file 2 — Supplementary file2 (TIF 500 KB) [file 10753_2023_1919_MOESM2_ESM.tif]

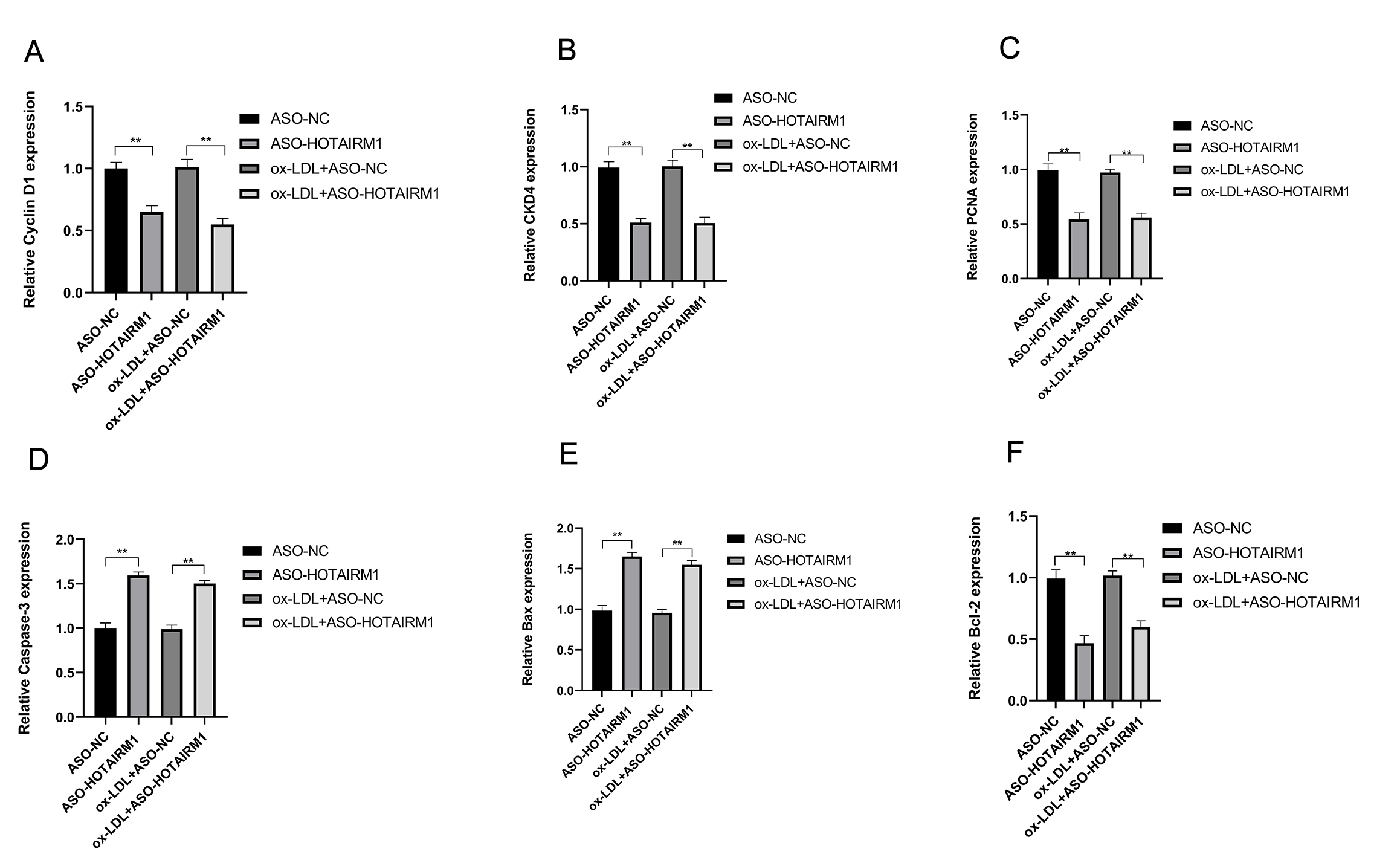

Supplement: Supplementary file 5 — Supplementary file5 (TIF 361 KB) [file 10753_2023_1919_MOESM5_ESM.tif]
